# Drug-resistant Mycobacterium tuberculosis among Nepalese patients at a tuberculosis referral center

**DOI:** 10.1371/journal.pone.0301210

**Published:** 2024-05-06

**Authors:** Arun Bahadur Chand, Ajaya Basnet, Bhagwan Maharjan, Ganesh Rai, Yadav Prasad Joshi, Lok Raj Bhatt, Bindu Sen, Shiba Kumar Rai

**Affiliations:** 1 Department of Microbiology, KIST Medical College and Teaching Hospital, Lalitpur, Nepal; 2 Department of Medical Microbiology, Shi-Gan International College of Science and Technology, Kathmandu, Nepal; 3 German Nepal Tuberculosis Project, Kathmandu, Nepal; 4 Department of Public Health, Manmohan Memorial Institute of Health Sciences, Kathmandu, Nepal; 5 Department of Dentistry, KIST Medical College and Teaching Hospital, Lalitpur, Nepal; 6 Department of Microbiology, Nepal Medical College Teaching Hospital, Kathmandu, Nepal; Lady Hardinge Medical College, INDIA

## Abstract

**Background:**

Multidrug-resistant tuberculosis (MDR-TB), characterized by isoniazid and rifampicin resistance, is caused by chromosomal mutations that restrict treatment options and complicate tuberculosis management. This study sought to investigate the prevalence of pre-extensively drug-resistant (pre-XDR) and extensively drug-resistant (XDR) tuberculosis, as well as mutation pattern, in Nepalese patients with MDR/rifampicin-resistant (RR)-TB strains.

**Methods:**

A cross-sectional study was conducted on MDR/RR-TB patients at the German Nepal Tuberculosis Project from June 2017 to June 2018. The MTBDRsl line probe assay identified pre-XDR-TB and XDR-TB. Pre-XDR-TB included MDR/RR-TB with resistance to any fluoroquinolone (FLQ), while XDR-TB included MDR/RR-TB with resistance to any FLQ and at least one additional group A drug. Mutation status was determined by comparing bands on reaction zones [*gyrA* and *gyrB* for FLQ resistance, *rrs* for SILD resistance, and *eis* for low-level kanamycin resistance, according to the GenoType MTBDRsl VER 2.0, Hain Lifescience GmbH, Nehren, Germany definition of pre-XDR and XDR] to the evaluation sheet. SPSS version 17.0 was used for data analysis.

**Results:**

Out of a total of 171 patients with MDR/RR-TB, 160 had (93.57%) had MTBC, of whom 57 (35.63%) had pre-XDR-TB and 10 (6.25%) had XDR-TB. Among the pre-XDR-TB strains, 56 (98.25%) were FLQ resistant, while 1 (1.75%) was SLID resistant. The most frequent mutations were found at codons MUT3C (57.14%, 32/56) and MUT1 (23.21%, 13/56) of the *gyrA* gene. One patient had SLID resistant genotype at the MUT1 codon of the *rrs* gene (100%, 1/1). XDR-TB mutation bands were mostly detected on MUT1 (30%, 3/10) of the *gyrA* and *rrs*, MUT3C (30%, 3/10) of the *gyrA*, and MUT1 (30%, 3/10) of the *rrs*.

**Conclusions:**

Pre-XDR-TB had a significantly higher likelihood than XDR-TB, with different specific mutation bands present in *gyrA* and *rrs* genes.

## Introduction

Tuberculosis (TB) stands as the second most prevalent infectious cause of death worldwide, claiming countless lives [1]. This disease, caused by the *Mycobacterium tuberculosis* complex (MTBC), spreads from person to person through airborne transmission [2,3]. Alarming statistics from 2019 reveal that a staggering 10 million individuals were afflicted by tuberculosis globally, resulting in 1.4 million deaths and 465,000 cases of MDR/RR-TB [4]. South-East Asia bears a substantial burden, accounting for 43% of the global impact [5]. In Nepal, around 117,000 people are affected by tuberculosis, which is also the seventh leading cause of death [6].

The development of antimicrobial resistance in *M. tuberculosis* stems from spontaneous mutations in various chromosomal genes, leading to altered interactions between anti-tuberculosis drugs and their intended targets [7]. For instance, resistance to fluoroquinolone (FLQ) arises from mutations in the *gyrA* or *gyrB* gene [8], while amikacin and kanamycin resistance are linked to mutational changes in the *rrs* gene and conformational alterations in the aminoglycoside acetyltransferase gene (*eis*) [9,10]. In developing countries with limited resources and high tuberculosis burdens, routine testing for resistance to fluoroquinolone *gyrA*, *gyrB*, and second-line injectable drugs (SLID) is often not feasible, potentially contributing to the spread of drug resistant *M. tuberculosis* strains [11]. Consequently, delayed drug susceptibility testing and the use of inappropriate regimens may heighten the risk of second-line drug (SLD) resistance [12].

The emergence of pre-extensive drug resistant (pre-XDR) and extensively drug resistant (XDR) *M. tuberculosis* isolates globally underscores the importance of molecular genotyping, which can aid in understanding tuberculosis transmission dynamics and identifying prevalent genotypes in specific geographic areas. Moreover, timely detection of pre-XDR-TB cases among multi-drug resistant (MDR)-TB patients is crucial for preventing treatment failure and implementing effective measures to curb the progression to XDR-TB [13].

The German Nepal Tuberculosis Project (GENETUP) conducted a survey in 2012 to gather information on pre-XDR-TB and XDR-TB. However, there was insufficient data available on these conditions and the specific gene responsible for drug-resistant tuberculosis in Nepal. As a result, the objective of this study was to determine both the prevalence of pre-XDR-TB and XDR-TB and the pattern of particular mutation bands related to FLQ and SLID resistance in clinical isolates obtained from Nepalese patients diagnosed with multidrug ‐ or rifampicin-resistant *M. tuberculosis*.

## Materials and methods

### Study design and population

This cross-sectional study was conducted at the German Nepal Tuberculosis Project (GENETUP), National Reference Laboratory, Kathmandu, Nepal, from June 2017 to June 2018 on patients diagnosed with MDR/RR-TB.

A total of 171 confirmed patients with MDR/RR-TB from various provinces across Nepal were selected for the study, including diverse genders, encompassing both males and females and age groups ranging from 12 to 75 years. MDR-TB cases were identified using both conventional drug susceptibility tests and the genotype MTBDRplus line probe assay, while RR-TB cases were diagnosed using GeneXpert. Sputum samples from 11 different sites across the country, including Rapid Test Center, Baglung Hospital, Team Hospital (Province: 4), Bhim Hospital, Lumbini Zonal Hospital (Province: 5), TB Nepal Provincial Hospital (Province: 6), Seti Zonal Hospital, Mahakali Zonal Hospital, Dandeldhura Hospital, and Bayelpata Hospital (Province: 7), were collected at the GENETUP National Reference Laboratory in Kathmandu, Nepal (Province: 3). The collected samples underwent SLD resistance testing, which included microscopic examination, decontamination, microbiological culture, and a line probe assay test at GENETUP National Reference Laboratory, Kathmandu, Nepal. The study included MDR/RR-TB Nepalese patients enrolled in the SLD resistance program of the National Tuberculosis Program, excluding those who had started SLD treatment (FLQs and aminoglycosides/cyclic peptides) during sample collection.

### Ethical approval

This study received approval from the Institutional Review Committee (IRC) of Shi-Gan Health Foundation in Kathmandu, Nepal (Reference number: 2074/12/10). Patient details were collected with the consent of each participant or their parent/guardian. The privacy of the data was protected by assigning a unique laboratory identity number to each participant.

### Data collection

Patient socio-demographic data was collected from the hospital record section, while information on pre-XDR-TB and XDR-TB cases, along with their specific mutation bands responsible for FLQ and SLID resistance, was gathered from the laboratory. Once complete and detailed information was obtained, it was verified, anonymized, and then entered and curated using Microsoft Excel 2010. Missing or ambiguous records were addressed through communication with healthcare workers involved in the process.

### Specimen processing and laboratory diagnosis

Out of a total of 171 cases, 126 were from direct sputum samples and 45 were from culture positive isolates. The GenoType MTBDRsl assay was performed to detect drug susceptibility test as well as MTBC and Mycobacterium other than tuberculosis (MOTT) following the standard protocol provided by the manufacturing company Hain Lifescience GmbH, Nehren, Germany [14].

#### Decontamination

After collecting the samples, they were transferred into appropriately labeled 50ml falcon tubes, with a volume of 3-5ml each, in preparation for further processing. The specimens underwent processing using a standard N-acetyl-L-cysteine-NaOH-sodium citrate digestion decontamination solution [15]. Subsequently, a DNA extraction was performed using 500μl of the re-suspended sample.

### Molecular analysis

- *DNA extraction*. DNA was extracted from both direct sputum specimens and cultured isolates of bacteria grown on solid Lowenstein-Jensen medium, using the GenoLyse® kit. The extraction followed the protocol provided by Hain Lifescience GmbH, Nehren, Germany [14].
- *Amplification and hybridization*. The amplification master mixture (45μl) was prepared in a PCR hood in a DNA-free room. Subsequently, the DNA sample (5μl) was added in a separate area within a class II biosafety cabinet. The amplification master mixture was prepared and the hybridization process was carried out according to the standard protocol outlined in the manufacturer’s instructions (Hain Lifescience GmbH, Nehren, Germany) [14].
- *Interpretation of results*. The assessment of resistance to second-line anti-TB drugs relied on analyzing the wild type and mutation band patterns. The examination of the *gyrA* and *gyrB* genes aimed to identify resistance to FLQs (e.g., ofloxacin or moxifloxacin), while the analysis of the *rrs* gene targeted cross-resistance to aminoglycosides/cyclic peptides antibiotics such as kanamycin, amikacin, capreomycin, and viomycin. Furthermore, the *eis* gene was examined to detect low-level kanamycin resistance [14].

### Statistical analysis

We utilized SPSS software (IBM SPSS version 17.0) for conducting statistical analyses. Descriptive statistics [mean (± SD), n, and %] were employed to depict the study variables. P-values < 0.05 were considered as statistically significant.

## Results

### Socio-demographic details of the studied sample

**Fig 1:**
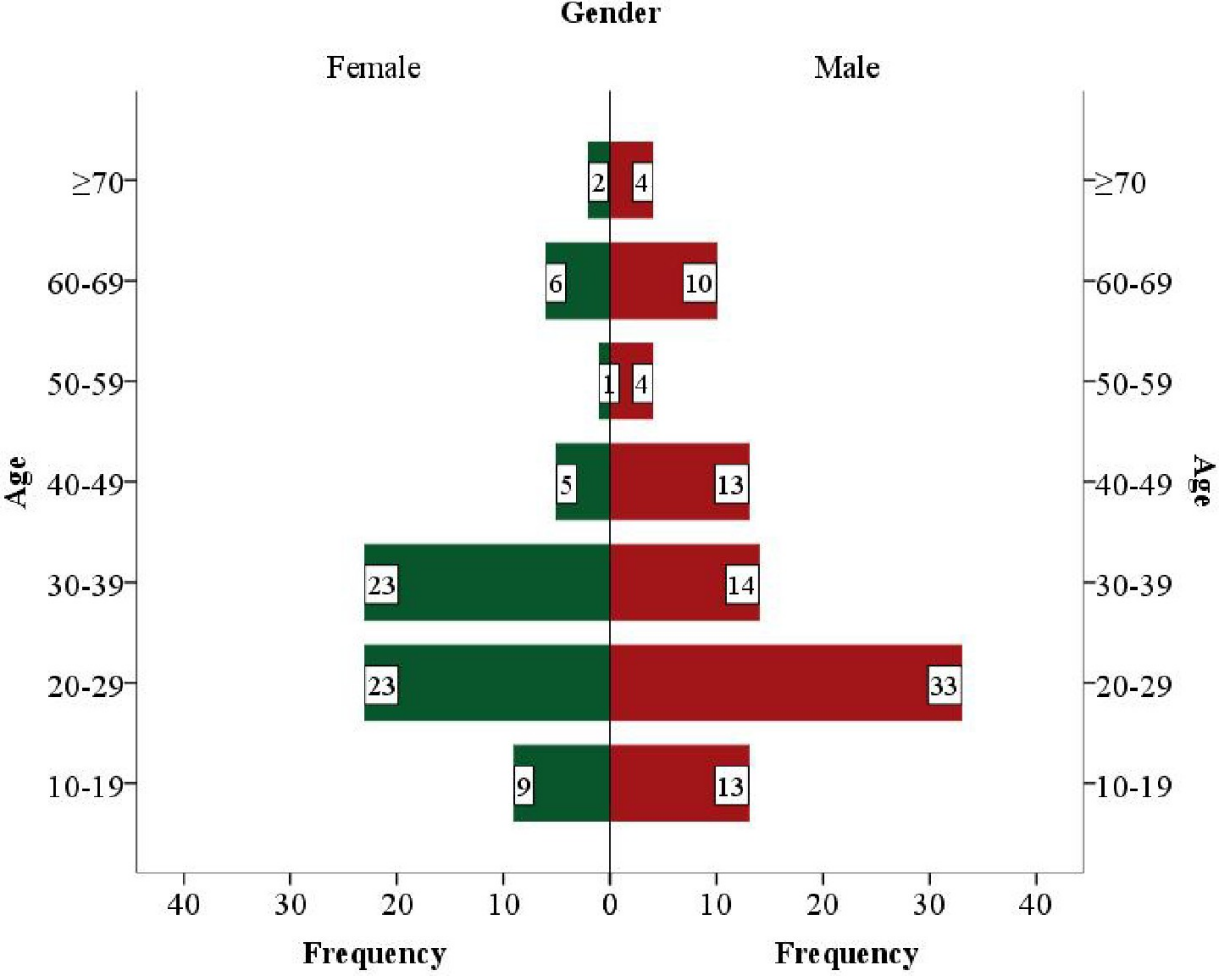
Age and gender based distribution of the patients infected with *M*. ***tuberculosis* complex.** In 160 MTBC positive cases, 91(56.88%) were male and 69(43.13%) were female participants. The highest number of male participants belonged to age 20–29 years whereas the age range for females was 20–29 years and 30–39 years.

Among a total of 171 samples, 160 (93.57%) were positive for MTBC and 11 (6.43%) were negative for MTBC (MOTT). The median [Interquartile (Q1-Q3)] age of patients with MTBC was 30 (22–40.75) years. There were 91 (56.88%) males with MTBC. In 160 MTBC positive cases, 126 (78.75%) [96 (76.19%) were smear positive and 30 (23.81%) were smear negative cases] were direct sputum specimens. Of 96 (76.19%) smear positives, 95 (98.96%. 95/96) were MTBC positive and 1 (1.04%, 1/96) was MTBC negative (MOTT) while in smear negative cases, 22 (73.22%, 22/30) were MTBC positives and 8 (26.67%, 8/30) were negatives (MOTT). However, 43 (95.5%, 43/45) of 45 cultures positive isolates tested positive for MTBC, while 2 (4.44%, 2/45) tested negative (MOTT) (Fig 1).

### Distribution of pre-XDR-TB and XDR-TB drug resistance cases

**Fig 2:**
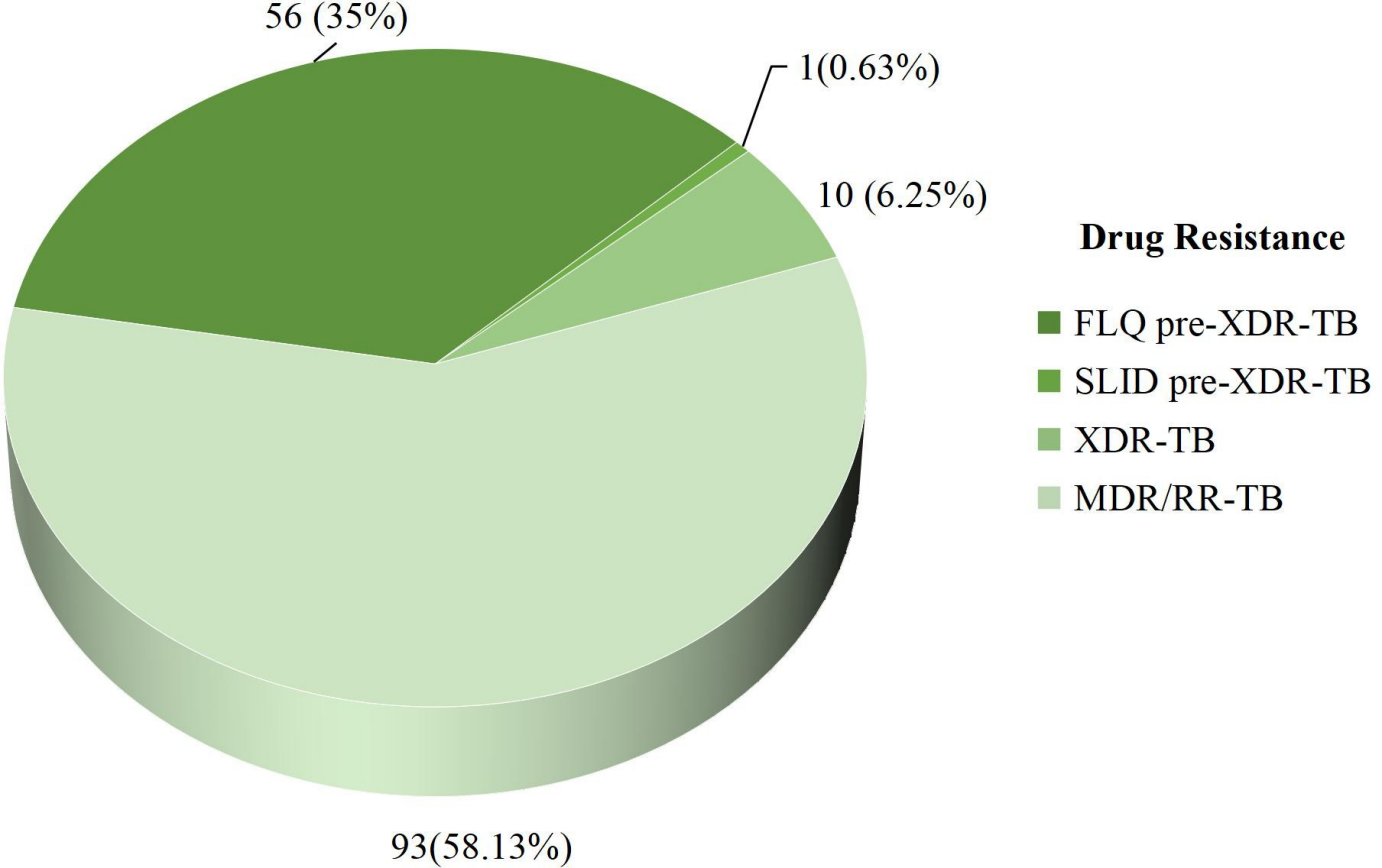
Diagram shows the distribution of pre-XDR-TB and XDR-TB drug resistance cases. This pie chart shows the prevalence of drug resistance patterns observed in cases of pre-XDR-TB and XDR-TB.

Among 160 MTBC positive cases, 56 (35.0%) were FLQ resistance pre-XDR-TB, 1 (0.63%) was SLID resistance pre-XDR-TB, and 10 (6.25%) were XDR-TB cases (Fig 2).

### Frequencies of mutations responsible for pre-XDR-TB and XDR-TB

Among 160 MTBC positive cases, 93 (58.13%) were susceptible to FLQ and SLID whereas 56 (35.0%) were resistance to FLQ (pre-XDR-TB) and 1 (0.63%) has resistance to SLID (pre-XDR-TB). The most frequently observed mutations were at codon MUT1 (23.21%, 13/56) and MUT3C (57.14%, 32/56) of *gyr*A gene (Table 1).

**Table 1:** Frequencies [n (%)] of mutations responsible for either FLQ or SLID resistance.

Drug	Mutation band	*gyrA* n = 56	*gyrB* n = 0	FLQ drug status
FLQ	*gyrA* MUT1	13(23.21)	0(0)	R
	*gyrA* MUT2	2(3.57)	0(0)	R
	*gyrA* MUT3A	1(1.79)	0(0)	R
	*gyrA* MUT3B	3(5.36)	0(0)	R
	*gyrA* MUT3C	32(57.14)	0(0)	R
	*gyrA* MUT3D	1(1.79)	0(0)	R
	*gyrA* MUT1 + MUT3A	1(1.79)	0(0)	R
	*gyrA* MUT 3B + MUT3C	1(1.79)	0(0)	R
	*gyrA* WT missing	2(3.57)	0(0)	R
**Drug**	**Mutation band**	***Rrs*** **n = 1**	***eis*** **n = 0**	**SLID status**
SLID	*rrs* MUT1	1(100)	0(0)	R

WT missing- Wild type probe missing FLQ- Fluoroquinolones, SLID- Second line injectable drug, R- Resistance.

Among 160 MTBC positive cases, 10 (6.25%) were resistance to FLQ and SLID (XDR-TB according to the previous definition). The most frequently observed mutations were at codon MUT1 (30%, 3/10) and MUT3C (30%, 3/10) of *gyr*A gene and at codon MUT1 (30%, 3/10) of the *rrs* gene (Table 2).

**Table 2:** Frequencies [n (%)] of mutations responsible both FLQ and SLID resistance.

Drug	Mutation band for FLQ and SLID				FLQ and SLID status	Total isolates with mutation n = 10
	*gyrA*	*gyrB*	*rrs*	*eis*		
FLQ and SLID	MUT1	WT missing	WT	WT missing	R	1(10)
	MUT1	WT	MUT1	WT	R	3(30)
	MUT3C	WT	WT	WT missing	R	1(10)
	MUT3C	WT	MUT1	WT	R	3(30)
	MUT3C	WT	MUT2	WT	R	1(10)
	MUT3C+ MUT1	WT	MUT1	WT	R	1(10)

WT- Wild type probe, WT missing- Wild type probe missing, R- Resistance, FLQ-Fluoroquinolone, SLID- Second-line injectable drug.

### Pre-XDR-TB and XDR-TB based on age and gender of the patients

In a total of 171 MDR/RR-TB cases, 6.43% (11/171) were MOTT, 35.63% (57/160) were pre-XDR-TB and 6.25% (10/160) were XDR-TB cases. In 160 MTBC positive cases, 66 (41.25%) were resistance to FLQ, the higher resistance was found in males 41 (25.63%) than females 25 (15.63%). In 11 (6.88%) SLID resistance cases, males 7 (4.38%) had more resistance cases than females 4 (2.50%). The age group 16–59 years had the highest FLQ resistance of 61 (38.18%) and the highest SLID resistance of 10 (6.25%). Fluoroquinolone resistance pre-XDR-TB 52 (91.23%, 52/57), SLID resistance pre-XDR-TB 1 (1.75%, 1/57), and XDR-TB 9 (90%, 9/10) resistance were all highest in the 16–59 years age group (Table 3).

**Table 3:** Distribution [n (%)] of pre-XDR-TB and XDR-TB based on age and gender of the patients.

Demographics		pre-XDR-TB n = 57		XDR-TB n = 10		Pearson Chi-Square(pre XDR or XDR TB)
		FLQ n = 56	SLID n = 1	FLQ n = 10	SLID n = 10	
Age group (years)	≤ 15: Children	2(3.51)	0(0)	0(0)	0(0)	0.55
	16–59: Adults	52(91.23)	1(1.75)	9(90)	9(90)	0.74
	≥60: Elderly	2(3.51)	0(0)	1(10)	1(10)	0.36
Gender	Male	35(61.40)	1 (1.75)	6(60)	6(60)	0.85
	Female	21(36.8)	0(0)	4(40)	4(40)	

SLID–Second line injectable drug, pre-XDR–Pre-extensively drug resistant, XDR–Extensively drug resistant.

The proportion of pre-XDR-TB patients in the children, adult, and elderly age groups is different from the proportion of XDR-TB patients. The proportion of pre-XDR-TB by gender is different from the proportion of XDR-TB (Table 3).

### Distribution of pre-XDR-TB and XDR-TB at province level

**Fig 3:**
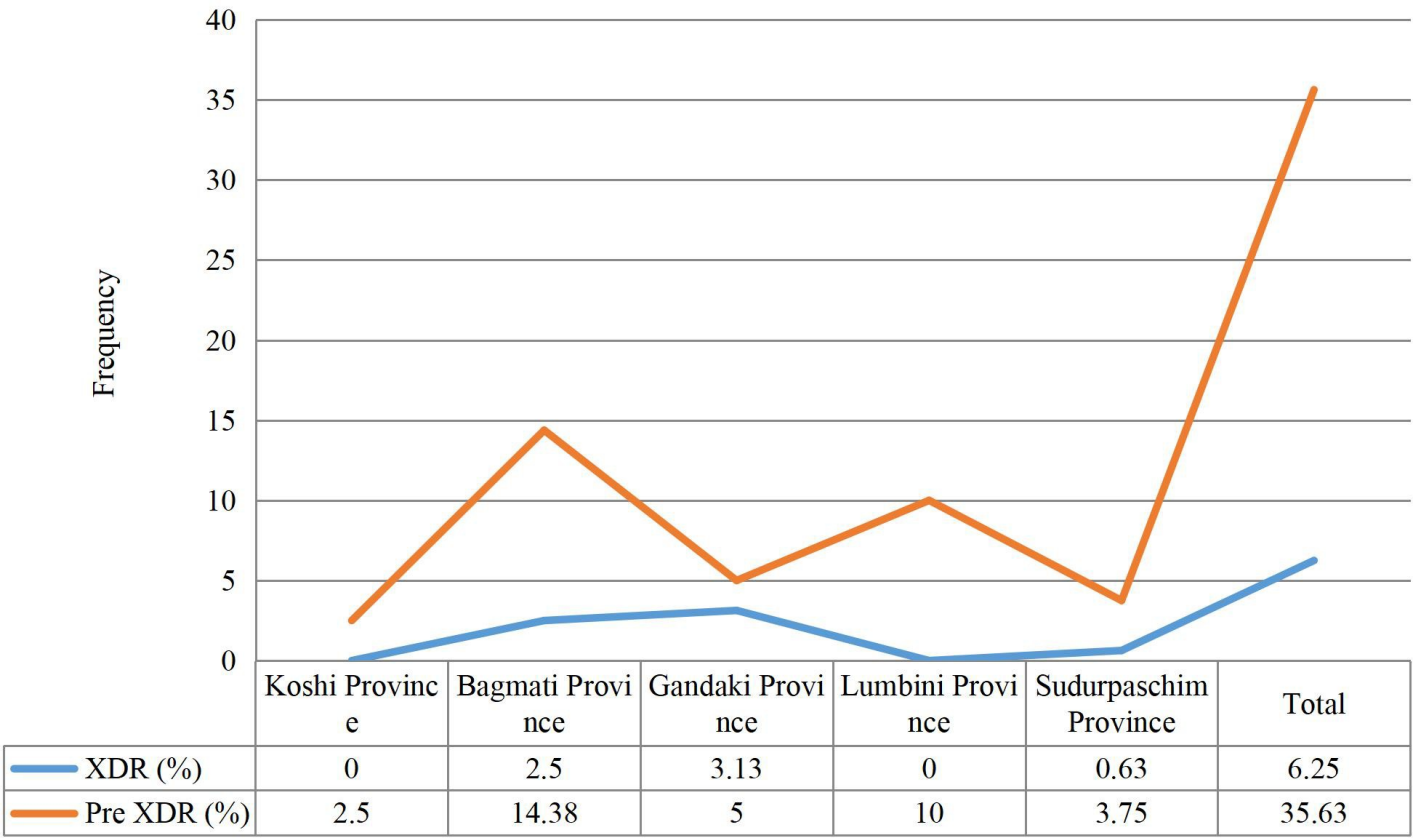
Pattern for pre-XDR-TB and XDR-TB cases. This line chart shows the pattern of pre-XDR-TB and XDR-TB across the provinces of Nepal, highlighting the provinces with the highest and lowest prevalence rates of pre-XDR-TB and XDR-TB.

In a total of 160 MTBC positive cases, the Bagmati and Gandaki provinces had the highest prevalence of pre-XDR-TB 23 (14.38%) and XDR-TB 5 (3.13%) cases respectively while the Koshi Province had the lowest prevalence of pre-XDR-TB 4 (2.50%) (Fig 3).

## Discussion

Tuberculosis, caused by MTBC, is an ancient disease [16]. Rapid molecular assays have emerged as a superior and more reliable method for detecting drug-resistant TB, surpassing conventional culture techniques in terms of time and specificity. A notable example is GenoTypeMTBDRsl version 2.0, an enhanced version of GenoTypeMTBDRsl version 1.0 that contains fewer mutations. This advanced test can detect molecular FLQ resistance involving *gyrA* and *gyrB*, as well as SLID resistance involving the *rrs* and *eis* genes [14]. Precisely diagnosing MDR-TB, pre-XDR-TB, and XDR-TB is crucial for interrupting transmission and selecting appropriate treatment options, including injectable drugs and FLQs. A thorough understanding of the drug resistant pattern plays a vital role in effectively managing pre-XDR-TB and XDR-TB cases.

This study revealed a higher prevalence of pre-XDR-TB (35.63%) and XDR-TB (6.25%). Similarly, a previous study conducted by GENETUP in 2012 reported a prevalence of 28% for pre-XDR-TB and 8% for XDR-TB [17]. In contrast, previous studies have shown divergent rates, with India reporting a pre-XDR-TB rate of 56%, China at 34%, and Bangladesh at 16% [13,18,19]. Similarly, multi-center studies of MDR-TB patients across eight nations and Poland found a comparable rate of XDR-TB, with 6.7% and 6.4%, respectively [20,21]. The inappropriate use of anti-TB drugs has contributed to the increased prevalence of pre-XDR-TB and XDR-TB patients, leading to the emergence of drug-resistant TB [22]. Specifically, the common use of FLQ in medical treatment has been linked to the increased prevalence of pre-XDR-TB. In this study, the prevalence of the *gyrA* MUT3C region associated with FLQ resistance was found to be the highest (57.14%, 32/56), while the *rrs* MUT1 region linked to SLID resistance showed a prevalence of 100% (1/1). For the specific mutation bands responsible for pre-XDR-TB, the MUT1 (30%, 3/10) and MUT3C (30%, 3/10) regions, associated with FLQ and SLID, respectively, had a considerable number of total isolates with specific mutations responsible for XDR-TB. A previous study in South Africa also observed frequent mutations in *gyrA* MUT1, *gyrA* MUT3C, and *rrs* MUT1, which align with the findings of our analysis regarding the frequency of specific bands on mutation regions [23].

Herein, the prevalence of FLQ resistance in pre-XDR-TB cases is significantly higher (98.25%, 56/57) compared to SLID resistance (1.75%, 1/57) in pre-XDR-TB cases. A study conducted in India also highlights the alarming global increase in FLQ resistance tuberculosis [11]. Another study by Ahmad et al. reported lower rate (52.7%) of FLQ resistance in MDR-TB patients [24]. In Delhi, India, a study revealed a notable prevalence of FLQ resistance among *M. tuberculosis* isolates, including drug-sensitive and multidrug-resistant strains [25]. The mechanism by which FLQ works is by blocking DNA gyrase, a crucial enzyme for bacterial DNA synthesis. *Mycobacterium tuberculosis* develops resistance to FLQ mainly due to mutations in DNA gyrase, which is composed of two A and two B subunits encoded by the genes *gyrA* and *gyrB*, respectively [26]. High level resistance to FLQ is often associated with mutations in the *gyrA* gene, while low-level resistance can be attributed to mutations in *gyrB*. In addition, another contributing mechanism involves efflux pumps that expel the drug from bacterial cells [27,28]. The inappropriate use of antimicrobial drugs, as well as the use of ineffective drug formulations and premature treatment interruption, can lead to drug resistant. This resistant can then be transmitted, particularly in crowded environments such as prisons and hospitals [29].

It Is interesting to note that a higher number of pre-XDR-TB and XDR-TB cases were diagnosed in the adult (16–59 years) age group. Independent studies in Bangladesh and India also reported an increased prevalence of pre-XDR-TB among the 21–30 and 18–25 age groups, respectively [13,18]. Moreover, the prevalence of FLQ and SLID resistance TB was notably higher in individuals aged 16–59, especially among males compared to females. The previous study on FLQ and SLID resistance did not specifically explore variations in drug resistance patterns with age and gender [30]. However, it is possible that within this age group, particularly among males, increased mobility, extensive environmental exposure, and outdoor activities might contribute to the higher prevalence of drug-resistant tuberculosis.

The study emphasizes the importance of timely detection of pre-XDR-TB and XDR-TB cases through efficient diagnostic tools to improve treatment strategies and align with global efforts to eradicate tuberculosis. However, a limitation of the study is the small data size specific to pre-XDR-TB, XDR-TB, and diagnostic facilities in GENETUP, as well as the ability of the MTBDRsl line probe assay to identify restricted mutations responsible for pre-XDR-TB, XDR-TB.

## Conclusions

This study found a higher prevalence of pre-XDR-TB and XDR-TB compared to previous studies. Notably, the prevalence of FLQ resistant cases was significantly higher than that of SLID within pre-XDR-TB. The most prevalent mutations were found in the *gyrA* gene at codons MUT1 and MUT3C and in the *rrs* gene at codon MUT1 were associated with SLID. This study highlights the importance of early diagnosis and prompt treatment for drug-resistant tuberculosis in Nepal.
